# Odd thyroid function tests results: interference versus non-compliance

**DOI:** 10.1186/1756-6614-6-S2-A37

**Published:** 2013-04-05

**Authors:** Krzysztof C Lewandowski

**Affiliations:** 1 Department of Endocrinology and Metabolic Diseases, Medical University of Lodz, Poland

## 

Despite the advent modern immunoassays, particularly with regards to TSH measurements, clinicians still encounter seemingly inexplicable results of thyroid function tests, for instance raised concentrations of free hormones (free T4 and/or free T3) in the setting of high, or at least not suppressed TSH concentrations. Though cases of TSH-oma or thyroid hormone resistance do exist, they are in fact rare, while some “strange” thyroid function tests results can be commonly explained either by a laboratory error or patient’s non-compliance with L-thyroxine treatment. In the latter case, a patient might deny any compliance problems, while a combination of raised TSH accompanied by an increased free T4 (±free T3) concentration is typically observed. This is usually a result of taking some extra doses of L-thyroxine prior to blood test, in order to catch up for previously missed doses. Confirmation of non-compliance is essential, as it avoids the need for lengthy and costly investigations in order to rule out malabsorption problems, assay interference or genuine cases of thyroid hormone resistance/TSH-oma. There are some strategies that help to demonstrate non-compliance, such as monitoring of thyroid function tests after controlled administration of high dose of L-thyroxine. An example of a case of a non-compliant hypothyroid patient after thyroidectomy for Graves’ disease will be presented, as well as a strategy that helped to elucidate this problem. I will also present the data on the efficacy of once weekly L-thyroxine administration in comparison to daily L-thyroxine administration protocol. Such approach might be sometimes applied in order to deal with some cases of persistent non-compliance with L-thyroxine treatment.

Nevertheless, there are cases where abnormal results of thyroid function tests persist, even though patient’s non-compliance has been ruled out. In such cases, assay interference, typically caused by the presence of heterophilic antibodies, must be suspected. These antibodies, usually directed against rodent antigens, are actually quite common, but most modern assays contain blocking antibodies that eliminate this problem in the majority of cases. If, however, the titre of such antibodies is extremely high, this may lead to abnormal test result. It should be mentioned that heterophilic antibody interference is not limited to results of thyroid function tests, but it may affect all other hormonal assays that involve the use of antibodies (e.g. sex steroid assays, etc) as well as other assays, for instance measurements of cardiac troponins. The likelihood of presence of high titre of heterophilic antibodies is increased in the setting of an augmented propensity for generation of autoantibodies, particularly in cases of coexistent rheumatoid disorders (such as rheumatoid arthritis). In cases of suspected assay interference, a clinician must decide in the first instance, whether obtained results fit the clinical picture. I shall endeavour to present a case of suspected heterophilic antibodies interference and I will also describe strategies to deal with such problems. The last, but not least I will also mention recently described cases of assay interference caused by the presence of macro-TSH particles, that may result in abnormal TSH measurements in a manner similar to macroprolactin.

